# Objective measurement of physical activity levels and patient-reported outcomes in cancer patients receiving immune-checkpoint inhibitors: a cross-sectional study

**DOI:** 10.1007/s00520-025-09824-9

**Published:** 2025-08-18

**Authors:** Rita Carrilho Pichel, Luísa Soares Miranda, Hugo Miguel Miranda, Susana Vale, João Queirós Coelho, Laura Pratas Guerra, Miguel Martins Braga, Gustavo Pinhol, Paula Fidalgo, Alexandra Araújo, António Araújo

**Affiliations:** 1 Centro Hospitalar Universitário de Santo António, Unidade Local de Saúde de Santo António, Serviço de Oncologia Médica, Porto, Portugal; 2 Oncology Research, Unit for Multidisciplinary Research in Biomedicine (UMIB), ICBAS-School of Medicine and Biomedical Sciences, University of Porto, Porto, Portugal; 3 Research Center in Physical Activity Health and Leisure (CIAFEL), Faculty of Sport, University of Porto, Porto, Portugal; 4 Laboratory for Integrative and Translational Research in Population Health (ITR), University of Porto, Porto, Portugal

**Keywords:** Physical activity, Cancer, Immune-checkpoint inhibitors, Accelerometer monitoring, Patient-reported outcomes, Quality of life

## Abstract

**Objectives:**

This study aimed to objectively assess physical activity levels in cancer patients treated with immune-checkpoint inhibitors (ICIs) and explore their association with patient-reported fatigue and quality of life (QoL).

**Methods:**

A cross-sectional study was conducted among adult patients with solid cancer *receiving* ICI treatment in the day hospital of our institution from March 26 to April 24, 2024. Physical activity levels were assessed using accelerometer monitors and the IPAQ questionnaire, while patient-reported fatigue and QoL were assessed with the FACIT-Fatigue and EQ-5D questionnaires, respectively. Descriptive statistics and between-group comparisons were performed, specifically between active and non-active patients, based on the WHO recommendation for healthy PA of ≥ 150 min/week of moderate-to-vigorous PA (MVPA), as well as between patients with or without problems in QoL domains.

**Results:**

A total of 23 patients were enrolled, with a mean age of 61 ± 12 years, 65.5% male. Eleven patients (47.8%) were active, with a mean MVPA of 22.5 ± 16.5 min/day based on objective measurements. Active and non-active subgroups were balanced for characteristics other than BMI. Based on 18 objective measurements, the mean MVPA time was 22.5 ± 16.5 min/day. Reported fatigue was higher than population norms, and 85.7% reported issues in QoL domains. Patients without problems in self-care or in usual activities reached higher PA levels, and patients without problems in mobility, self-care, or pain/discomfort reported less fatigue and a higher index of global health.

**Conclusion:**

Less than half of ICI-treated patients follow the recommendation for healthy PA. Patients engaging in more MVPA may have less problems in self-care and in usual activities. It reinforces the need to promote PA during immunotherapy.

## Abstract (plain language summary)

Cancer patients receiving chemotherapy often experience fatigue and reduced physical activity levels, but little is known about the physical activity levels of those receiving immunotherapy with immune-checkpoint inhibitors. Emerging research suggests that exercise might enhance the results of immunotherapy.

Our study aimed to measure physical activity levels during immunotherapy and examine their relationship with patient-reported fatigue and quality of life. We found that fewer than half of patients met physical activity recommendations and that patients who were more physically active reported fewer problems in self-care and usual activities.

These findings highlight the need to encourage physical activity in patients undergoing immunotherapy and may guide future research on improving their quality of life.

## Introduction

### Background

Fatigue is one hallmark symptom of cancer and can significantly impair patients’ quality of life (QoL) [1]. Notably, physical activity is significantly decreased in cancer patients with advanced disease and in those undergoing chemotherapy for early disease [2–6]. A large body of evidence concluded that exercise (defined as a planned, structured, and repetitive physical activity) is beneficial for cancer patients, for instance, improving cancer-related fatigue and health-related QoL [7–10].

Immunotherapy, especially with immune-checkpoint inhibitors (ICI), has changed the paradigm of cancer treatment. ICI are a class of drugs, first approved in 2011, constituted by monoclonal antibodies that block the interaction of immunoregulatory proteins and effector T cells, enhancing the immunological response against cancer [11–13]. Emerging preclinical data unveiled how immunotherapy efficacy depends on systemic immunity and how exercise may influence the immune system and enhance response to ICI [14–17]. Clinical evidence about physical activity or exercise effects among patients undergoing ICI therapy remains limited, yet it points out that physical activity may also help reduce treatment-related toxicities such as fatigue in this population. [18, 19] Moreover, there is a gap in the epidemiologic assessment of physical activity among patients receiving ICI.

Thus, we hypothesize that, among cancer patients treated with ICI, fatigue might be prevalent and physical activity levels might be reduced (as previously described for chemotherapy), which may compromise immunotherapy efficacy, and we expect that those who accomplish goals for physical activity may report less fatigue and better QoL.

### Objectives

Considering the recent rapid expansion of ICI indications and the potential of physical activity and exercise to optimize its efficacy and tolerability, our goal is to objectively assess the physical activity levels of cancer patients receiving ICI treatment and examine their correlation with patient-reported fatigue and QoL.

We hope our findings will help renew interest and drive further investment in physical activity promotion and adjuvant exercise interventions for this patient population.

## Methods

### Study design and setting

We conducted a cross-sectional observational analysis of physical activity levels and patient-reported fatigue and QoL in the day hospital of a tertiary university hospital center, for approximately 1 month.

### Participants selection

We screened our polyvalent day hospital for admissions for ICI treatment. We considered eligible ambulatory adult patients (≥ 18 years old) with solid cancer diagnosis who received at least one dose of ICI (pembrolizumab, nivolumab, ipilimumab, avelumab, or atezolizumab) administered intravenously in the day hospital of our institution. Patients who were unable to use the accelerometer or to complete questionnaires due to language or cognitive barriers were excluded.

### Physical activity levels assessment

Physical activity was objectively assessed with an Actigraph GT3M accelerometer (Manufacturing Technology, Fort Walton Beach, FL). Two investigators (LSM and RP) personally delivered and explained the use of the accelerometers to participants [20]. Accelerometers represent an accurate alternative to physical activity questionnaires, to monitor and measure levels of physical activity [6, 21, 22]. The accelerometer was firmly adjusted at the patient’s right hip by an elastic waist belt under their clothing. Participants were instructed to attach the accelerometer when they awoke and to remove it when they went to bed. Participants were asked to maintain usual activities. Participants were required to wear the accelerometer for a minimum of 5 (and a maximum of 7) consecutive days and at least 10 h per day. For participants who provided recordings for more than 5 consecutive days, only the data from the final 5 days, which included 2 weekend days, were considered. Data were analyzed per day by an experienced researcher using ActiLife5 LITE software (Actigraph, Pensacola, FL, USA). PA free-living quantity (at least within frequencies, ~ 2–4 Hz) and predefined epoch and sample rate were 15 s and 30 Hz, respectively. Average intensity (counts min^−1^) and counts were transformed into time (average min day^−1^ and total min week^−1^) engaged in either physical inactivity or light, moderate, and vigorous PA using the cutoffs proposed by Freedson et al. Moderate-to-vigorous physical activity (MVPA) was defined as ≥ 1952–5724 counts min^−1 ^[23]. If a patient forgot to use the accelerometer for 1 day, we calculated and used his daily mean time of MVPA to estimate the weekly MVPA.

Additionally, we applied a validated questionnaire; the International Physical Activity Questionnaire—Short Form (IPAQ-SF), ‘‘last week’’ version was used. IPAQ reports separately vigorous-intensity PA, moderate-intensity PA, and walking in terms of frequency and duration of each specific type of activity, in the past 7 days. This instrument also reports the time spent sitting in an ordinary weekday and weekend day. Both categorical and continuous indicators of PA are possible from the IPAQ short version. Validity and reliability data from 12 countries (including Portugal) have shown that IPAQ has comparable validity and reliability to CSA monitor and to other self-report PA measures [24]. According to the guidelines for data processing and IPAQ analysis (IPAQ, 2005), total PA can be expressed as MET-min/week (metabolic equivalent), by weighting the reported minutes per week, in each activity category, by the metabolic equivalent specific to each activity. The daily duration of each intensity of physical activity (moderate and vigorous) and walking (i.e., light physical activity) was computed multiplying the number of days per week by the time per day in each intensity. After that, the results for each physical activity intensity and walking were divided by 7 to get the mean values per day (i.e., light physical activity/day, moderate physical activity/day, and vigorous physical activity/day). Additionally, moderate and vigorous physical activity per day was summed to get moderate to vigorous physical activity. We followed Guidelines for Data Processing and Analysis of the IPAQ available at https://www.org.sites.google.com/view/ipaq/score.

To analyze the adherence to World Health Organization (WHO) guidelines on physical activity we assessed the accumulation of at least 150 min/week of MVPA or 75 min/week of vigorous activity [25]. These apply to adults and older adults with chronic conditions, including cancer survivors. Participants were classified as *active* or if they accomplished the physical activity recommendations. If this information was missing from accelerometer monitoring it could be retrieved from IPAQ-SF, and this was specified.

We have also collected self-monitor, daily walking distance, and data from patients’ own monitor devices monitors (ex. iPhone steps count).

### Patient-reported fatigue and quality of life

Fatigue and QoL were assessed using questionnaires validated for the Portuguese language and the cancer population. Permission to use the questionnaires for the purpose of this study was granted. The investigators (RP and LSM) personally explained and delivered the questionnaires to participants. Whenever possible, questionnaires were completed online using a form (Microsoft Forms©2024) accessed through a QR code or web link. When necessary, participants used the original paper version of the questionnaires. Help of a family member or investigator was allowed.

Fatigue was evaluated with Functional Assessment of Chronic Illness Therapy-Fatigue Scale (FACIT-Fatigue, Portuguese version 4). The FACIT Measurement System is a collection of health-related QoL (HRQOL) questionnaires that assess multidimensional health status in people with various chronic illnesses, specifically cance [21, 26]. FACIT-Fatigue is a 13-item patient-reported outcome instrument that was designed to assess fatigue-related symptoms and impacts on daily functioning. Item scores can range from 0 (“not at all”) to 4 (“very much”), and the total score from 0 to 52; *lower scores indicate greater fatigue*. The recall period for each item is the past 7 days. We followed the scoring document of instructions of the FACIT-Fatigue available at https://www.org/facit.org/measures/facit-fatigue. For subgroup comparison and interpretation purposes, we used the cutoff of 40 to stratify the patients that had more fatigue than the general population. This was based on the mean estimates of 43 for USA and Germany general populations, and the minimum meaningful difference of 3 points [27–29].

Quality of life was evaluated with EQ-5D-3L (Portuguese © 1997 EuroQol Group Versão Portuguesa, 1997, 2013.EQ-5D v2. Centro de Estudos e Investigação em Saúde da Universidade de Coimbra), respectively [30]. First, patients had to choose among three sentences (scored from 1 to 3) the better description of each of five domains of QoL: Mobility, Self-care, Usual activities, Pain/Discomfort, and Anxiety/Depression. In the second part, the global health index, patients were asked to grade their health in that day in a scale from 0 (“the worst health they can imagine”) to 100 (“the best health they can imagine”). We followed EQ-5D-3L User Guide for scoring and interpretation. It is available at https://www.org/euroqol.org/wp-content/uploads/2023/11/EQ-5D-3LUserguide-23–07.pdf.

### Covariates

Demographic and health characteristics were evaluated with a self-administered questionnaire, which contained questions on demographics. Cancer site and staging, treatment characteristics, body mass index (BMI) blood cell count, and comorbidities were obtained through hospital records. Cancer staging was defined according to the eighth edition of the AJCC staging system.

### Data protection

The questionnaires were answered through Microsoft Forms©2024 in a form created in the institutional account of the principal investigator (RP). The main study database was stored in the institutional OneDrive, protected with a security code, and it could be downloaded to the investigators own computers. As specified in the institutional Data Protection Form, data from the study population was safely and respectfully handled by the study team.

### Ethical concerns

Informed consent was obtained, and confidentiality of data was maintained throughout all stages of collection and results dissemination. This study did not interfere with therapy prescription or with the number of visits to the hospital, and it did not have direct costs or compensations to the patient. “Time toxicity” was diminished by explaining and applying the questionnaires and accelerometer at the day hospital session, while awing for pre-treatment results or during the immunotherapy infusion.

### Bias concerns

Selection bias may be present because of limited availably of accelerometers and the investigators’ capacity to approach all eligible patients in the period of the study. Inclusion was blind from the patients’ characteristics apart from the name of the ICI he was prescribed. Additionally, investigators sought to recruit eligible patients on different weekdays to enhance the heterogeneity of periodic therapeutic protocols.

Recall bias was diminished by using objective measurements and questionnaires referring to a recent and short period of time (the previous 7 days).

The investigator that analyzed the accelerometer data (SV) was blinded for the participants’ characteristics, and the investigators that collected the data by applying the questionnaires and accelerometer (RP and LSM) were not the usual Medical Oncologist of the participants.

### Study size

We have estimated that in 1-month period we would find approximately 150 eligible patients receiving ICI in the day hospital agenda, and this would allow us to find the proportion of *active* patients with a confidence interval of 95%. The final study size was limited by the day hospital schedule and the availability of accelerometers.

### Statistics

Categorical variables were reported as count and percentage and between-group comparisons were conducted using Chi-square test or Fisher’s exact test, as appropriate. For continuous variables, distribution was assessed with Shapiro–Wilk test and histogram, except for “time from diagnosis”, “time beginning of ICI”, and “lymphocyte count”, which followed a non-normal distribution; for other continuous variables normal distribution was assumed, and they were reported as mean and standard deviation and between-group comparisons were done using *t* test. Correlations were tested with Spearman’s correlation coefficient. IBM SPSS Statistics 29 software was used for statistical analysis. A *p* value < 0.05 was considered statistically significant and, unless otherwise specified, we used bilateral significance.

## Results

### Selection and description of participants

**Fig. 1:**
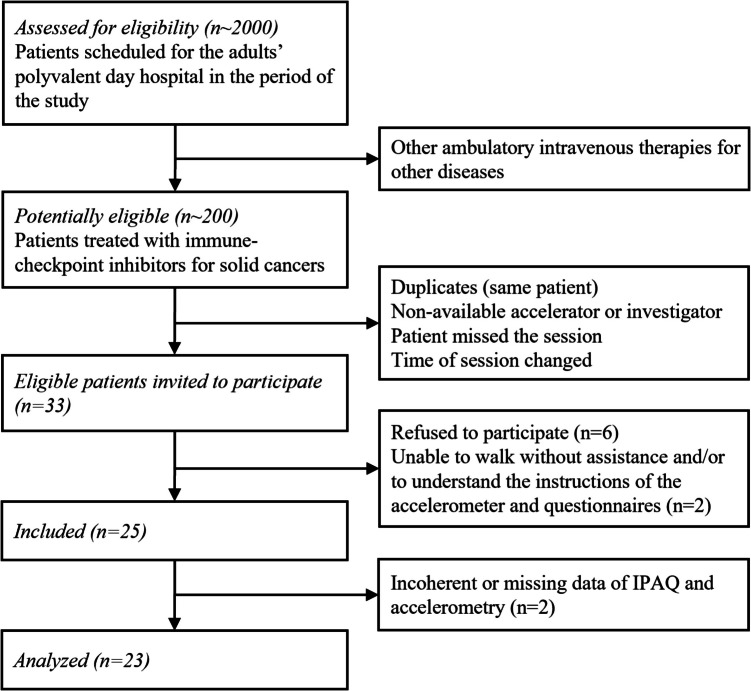
Flow diagram of selection. IPAQ International Physical Activity Questionnaire

Between March 26 and April 24, 2024, of 33 eligible patients invited to participate, 23 were selected. The selection flow diagram is represented in Fig. 1 and the characteristics of study participants and the comparison between *active* and *non-active* patient groups are presented in Table 1.

**Table 1:** Patients’ demographics and cancer and treatment characteristics. *N* = 23

			Active				Total		*p*
			Yes		No				
Gender, *n* %		Female	3	27.3%	5	41.7%	8	34.8%	0.667
		Male	8	72.7%	7	58.3%	15	65.2%	
Age, year, mean ± SD			60	± 11	62	± 13	61	± 12	0.699
Residence, *n* %	Custoias		1	9.1%	0	0.0%	1	4.3%	0.687
	Gondomar		1	9.1%	0	0.0%	1	4.3%	
	Porto		2	18.2%	4	33.3%	6	26.1%	
	Rio Tinto		2	18.2%	2	16.7%	4	17.4%	
	Sao Mamede Infesta		0	0.0%	1	8.3%	1	4.3%	
	São Pedro da Cova		0	0.0%	1	8.3%	1	4.3%	
	Valbom		1	9.1%	1	8.3%	2	8.7%	
	Non specified		4	36.4%	3	25.0%	7	30.4%	
Primary tumor site, *n* %		Breast	3	27.3%	4	33.3%	7	30.4%	0.813
		Head and neck	1	9.1%	1	8.3%	2	8.7%	
		Kidney	1	9.1%	2	16.7%	3	13.0%	
		Lung	3	27.3%	4	33.3%	7	30.4%	
		Other	3	27.3%	1	8.3%	4	17.4%	
Initial staging group (AJCC 8th ed.)		II	2	18.2%	4	33.3%	6	26.1%	0.662
		III	3	27.3%	2	16.7%	5	21.7%	
		IV	6	54.5%	6	50.0%	12	52.2%	
Immune-checkpoint inhibitor, *n* %		Atezolizumab	2	18.2%	2	16.7%	4	17.4%	0.844
		Avelumab	1	9.1%	1	8.3%	2	8.7%	
		Cemiplimab	0	0.0%	1	8.3%	1	4.3%	
		Durvalumab	1	9.1%	0	0.0%	1	4.3%	
		Nivolumab	1	9.1%	1	8.3%	2	8.7%	
		Pembrolizumab	6	54.5%	7	58.3%	13	56.5%	
Time from beginning of ICI, months, median IQR			6.3	2.8–13.1	5.1	2.8–14.2	5.6	2.8–13.5	0.756
Context/intent, *n* %		(Neo)adjuvant	3	27.3%	3	25.0%	6	26.1%	1.000
		Palliative	8	72.7%	9	75.0%	17	73.9%	
Concomitant chemotherapy, *n* %			3	27.3%	3	25.0%	6	26.1%	0.901
Previous chemotherapy, *n* %			8	72.7%	8	66.7%	16	69.6%	0.752
Previous radiotherapy, *n* %			1	9.1%	3	25.0%	4	17.4%	0.315
Previous surgery, *n* %			3	27.3%	2	16.7%	5	21.7%	0.538
BMI, kg/m^2^, mean ± SD			21.82	± 2.18	25.95	± 4.16	24.18	± 3.98	0.014*
Seric albumin, g/dL, mean ± SD			4.1	± 0.3	4.1	± 0.3	4.1	± 0.3	0.956
Hb, g/dL, mean ± SD			12.7	± 1.9	12.4	± 2.5	12.5	± 2.2	0.747
Lymphocytes,/μL, median IQR			1610	1048–1802	1360	930–2180	1515	1012–1870	0.821
Platelets,/μL, mean ± SD			216,100	± 55,806	213,916	± 66,224	214,909	± 60,276	0.935

The median age of participants was 61 ± 12 years (range 33–84). Most participants were male (*n* = 15, 65.5%), had lung or breast cancer (*n* = 7, 30.4% each), were diagnosed with advanced stage disease (*n* = 12, 52.2%), and were receiving palliative systemic treatment (*n* = 17, 73.9%). All participants were receiving ICI at the time of the study, for a median period of 5.6 months (IQR 2.8–13.5) and a mean of 14 ± 12 cycles. The most used ICI was pembrolizumab (*n* = 13, 56.5%).

### Physical activity measurement

Based on 23 patients’ assessments (18 objective measurements and five patients reported physical activity), 11 patients (47.8%) accomplished the recommendation for healthy physical activity (≥ 150 min/week of MVPA; zero patients did > 75 min/week of vigorous activity) being classified as *active*. *Active* patients had lower BMI (21.58 ± 2.30 vs. 25.47 ± 4.06, *p* = 0.014) than *non-active* patients, and there was a negative correlation between MVPA time with BMI (*ρ* = − 0.564, *p* = 0.018). No other statistically significant differences were observed between *active* and *non-active* participants (Table 1).

Based on 18 objective measurements, the mean MVPA time was 22.5 ± 16.5 min/day (157.7 ± 115.6 min/week) and seven patients (38.9%) accomplished the recommendation for healthy physical activity.

### Physical activity and patient-reported outcomes association

Based on reports from 21 patients, the mean fatigue score was 37.1 ± 8.3 (range: 22–50), with ten patients (50.2%) experiencing greater fatigue than the general population mean. Fatigue was significantly higher among patients with anemia, defined as Hb < 12 g/dL (FACIT-Fatigue score: 32.5 ± 7.7 vs. 40.4 ± 7.4, *p* = 0.039). No statistically significant differences in fatigue were observed between *active* and *non-active* patients (39.5 ± 5.6 vs. 35.3 ± 9.7, *p* = 0.289) or between patients receiving concomitant chemotherapy and those who were not (33.0 ± 8.4 vs. 38.1 ± 8.3, *p* = 0.287).

The mean global health index was 67.5 ± 20.6 (range: 30–100), and 18 patients (85.7%) reported experiencing problems in at least one domain of QoL. The most affected QoL domains were Pain/Discomfort (*n* = 13, 61.9%) and Mobility and Usual Activities (*n* = 11, 47.6% each).

Patients who reported no problems in self-care or usual activities had higher step counts and MVPA time compared to those with problems in these QoL domains (Table 2). Additionally, patients who reported no problems in mobility, self-care, or pain/discomfort experienced less fatigue and had a higher global health index than those with problems in these domains (Table 2). These and other subgroup comparisons between patients with or without problems in each QoL domain are detailed in Table 2.

**Table 2:** Comparison of physical activity measures and patient-reported outcomes, between patients with or without problems in the five domains of quality of life, assessed with the first part of the questionnaire EQ-5D-3L

	*1. Problems in mobility?*								
	*No (1 pt)*				*Yes (2–3 pts)*				*p*
	*n*	Mean	±	SD	*n*	Mean	±	SD	
MVPA time, min/day, *n* = 16	8	22.8	±	12.0	8	19.2	±	19.4	0.663
Steps count, per day, *n* = 16	8	4557	±	1431	8	3529	±	3264	0.434
FACIT-Fatigue score, *n* = 19	10	42.0	±	5.8	9	31.6	±	7.4	0.004*
Global health index, *n* = 20	10	77.5	±	13.0	10	57.5	±	22.5	0.029*
	*2. Problems in self-care?*								
MVPA time, min/day, *n* = 16	14	23.7	±	14.8	2	2	±	1.1	0.032*^a^
Steps count, per day, *n* = 16	14	4546	±	2230	2	526	±	376	< 0.001*
FACIT-Fatigue score, *n* = 19	16	37.6	±	8.2	3	34	±	10.4	0.506
Global health index, *n* = 20	17	71.8	±	19.3	3	43.3	±	5.8	0.023*
	*3. Problems in usual activities?*								
MVPA time, min/day, *n* = 16	9	27.1	±	14	7	13.1	±	15	0.037*^a^
Steps count, per day, *n* = 16	9	5180	±	1984	7	2582	±	2419	0.033*
FACIT-Fatigue score, *n* = 19	9	42.5	±	5.8	10	32.1	±	7.2	0.003*
Global health index, *n* = 20	10	77	±	13.8	10	58	±	22.5	0.035*
	*4. Pain/discomfort?*								
MVPA time, min/day, *n* = 16	4	18.4	±	7.7	12	21.9	±	17.8	0.605
Steps count, per day, *n* = 16	4	4052	±	1013	12	4040	±	2862	0.994
FACIT-Fatigue score, *n* = 19	8	41.5	±	4.3	11	33.8	±	9.2	0.029*
Global health index, *n* = 20	8	81.2	±	16.2	12	58.3	±	18.4	0.010*
	*5. Anxiety/depression?*								
MVPA time, min/day, *n* = 16	9	21	±	15.6	7	21	±	17	0.994
Steps count, per day, *n* = 16	9	4066	±	2682	7	4014	±	2436	0.968
FACIT-Fatigue score, *n* = 19	11	38.5	±	7.2	8	35.1	±	9.9	0.406
Global health index, *n* = 20	12	72.9	±	19.9	8	59.4	±	20.1	0.155

*MVPA* moderate to vigorous physical activity

^*a*^Unilateral *p* value was used

Self-monitor data in our sample was scarce and heterogeneous and did not retrieve additional significant results.

## Discussion

### Interpretation and historical comparison of main results

This study first measured the PA levels of cancer patients undergoing ICI treatment. We observed a mean MVPA time of 22.5 ± 16.5 min/day, under the recommendation for healthy physical activity [25]. The proportion of patients who did not follow this recommendation was high: 38.9% based on accelerometer monitoring only, and 47.8% if missing data was complemented with IPAQ results. Compared to Portuguese older adults population (> 65 years old, with a mean age 74 vs. 61 in our study), our study observed lower MVPA time (23 vs. 26 min/day) in cancer patients treated with ICI [31]. It would be interesting to explore in longitudinal studies, with larger homogeneous samples, if other immune-related adverse events (such as hypothyroidism and arthritis), were present and how to they correlate with physical activity levels.

In our sample of ICI-treated patients, FACIT-Fatigue mean scores (37 ± 8) were clinically meaningfully lower than general population norms (44 ± 8 and 44 ± 9, USA and Germany, respectively), [28, 29] and marginally higher than patients undergoing chemotherapy (34 ± 11), [2] suggesting that cancer patients treated with ICIs may feel more fatigue than general population and slightly less fatigue than those treated with chemotherapy. Although we failed to prove that there was statistically significant difference between *active* and *non-active* patients and between patients treated with concomitant chemotherapy and those who were not, the numerical difference of > 3points in FACIT-Fatigue score would be clinically meaningful and its direction is coherent with previous findings that *active* patients experience less fatigue and that the association with chemotherapy causes more fatigue [32]. Among non-anemic cancer patients, we observed similar patient-reported fatigue to previously described in literature for those treated with chemotherapy (*n* = 113, FACIT-Fatigue score: 40.0 ± 9.8) [28, 33]. Apart from hemoglobin level, other studies showed that interleukin-6 (IL-6) had influence on fatigue [34]. By its hand, exercise modulates IL-6 in a complex way, which may partially explain its benefits on cancer fatigue [35]. Recent feasibility studies have been conducted to test physical activity or exercise interventions to reduce fatigue in patients undergoing ICI treatment [36, 37].

The ICI-treated patients’ perception of global health was 67.5 in a 0–100 scale and a large percentage (85.7%) reported having problems in some domains of QoL. Interestingly, patients without problems in self-care or in usual activities spend more time doing MVPA, and patients without problems in mobility, self-care, or pain/discomfort reported less fatigue and a higher index of global health. As we explain in the study limitations subsection, due to the cross-sectional design, we cannot establish causality.

It would be important to prospectively assess if exercise guidance was being provided to each patient receiving ICI. The authors agree that exercise promotion was lacking for average patients, as reported for chemotherapy [38].

### Future perspectives

Lately, preclinical evidence pointed out that exercise may improve anti-tumor immunity, and higher physical activity levels at the start of ICI treatment were related to lower risk of severe immune-related adverse effects [14, 19, 39, 40]. Given this preliminary evidence and the results of our study, we hope to expand our sample and complement this cross-sectional study with longitudinal data to add knowledge about the interference of physical activity in the efficacy and safety of ICIs. Further prospective clinical studies are necessary to validate this potentially promising synergy.

Physical activity promotion should be assured as part of gold standard care, and patients’ education about the potential synergism between exercise and immunotherapy could help improve adherence.

### Generalizability

Our sample is representative of the growing indications of ICIs, having represented most used ICIs in Europe and the most prevalent cancers for which ICIs are currently indicated (such as lung cancer and early triple negative breast cancer). It is mostly representative of Porto city center and Gondomar, because of the refusal of patients who lived far from the hospital, for whom it was most inconvenient to return the accelerometer. The referral population of our hospital, from the national health system, downtown located, has a low socioeconomic background and many comorbidities that might have influenced results.

### Limitations

The cross-sectional design with convenience sample does not allow us to make causal inferences. Specifically, about physical activity, the fact that we do not know the baseline level of physical activity does not allow us to stratify accordingly or to see reduction/variation over the course of therapy. In our small sample there was no significant correlation between time of ICI and physical activity times.

By excluding patients who had cognitive or physical barriers to fulfill the assessments of the study we could have overestimated the percentage of *active* patients and physical activity times. The fact that accelerometers do not permit assessing water activities and they are limited in other activities, such as cycling, rowing, and some free weight exercises, may in turn underestimate it [41].

The size and heterogeneity of our sample limit the conclusions. Short professional time dedicated to investigational purposes and lack of specific funding justify this.

## Conclusion

This study revealed that less than half of cancer patients treated with immunotherapy in our institution reached the time goal of healthy physical activity. It reinforces the need to promote healthy physical activity by suggesting that those who spend more time in MVPA report less problems in *self-care* and in *usual activities*. Apart from its design and size limitations, this study was pioneer in objectively measuring physical activity in patients treated with ICI and once more it calls for attention for patient-reported fatigue with ICI.

We believe our results should drive further clinical investigation about this topic and reinforce the need to assess and assure healthy physical activity levels in clinical practice and as standard supportive care of Oncology trials, to improve QoL and global health results. Additionally, our data may be useful for clinicians to educate and motivate patients to change their health behaviors.

## Data Availability

Upon justified requirement, the raw data supporting the conclusions of this article will be made available by the authors, without undue reservation.
